# Light-Up of Rhodamine Hydrazide to Generate Emissive Initiator for Polymerization and to Afford Photochromic Polypeptide Metal Complex

**DOI:** 10.3390/polym9090419

**Published:** 2017-09-05

**Authors:** Jhen-Yan Gao, Wen-Chih Huang, Pei-Yi Huang, Cheng-Yu Song, Jin-Long Hong

**Affiliations:** Department of Materials and Optoelectronic Science, National Sun Yat-Sen University, Kaohsiung 80424, Taiwan; M033100005@student.nsysu.edu.tw (J.-Y.G.); M043100042@student.nsysu.edu.tw (W.-C.H.); M033100043@student.nsysu.edu.tw (P.-Y.H.); M033100058@student.nsysu.edu.tw (C.-Y.S.)

**Keywords:** aggregation-induced emission, polypeptide, metal ligand charge transfer, rhodamine, secondary structure, photochromic

## Abstract

Ring-opening polymerization (ROP) of cyclic peptide monomer of γ-propargyl-l-glutamate *N*-carboxyanhydride (PLG–NCA) was originally initiated by non-emissive, ring-close rhodamine 6G hydrazide (R-C). However, instantaneously after adding PLG–NCA to R-C, the spirolactam ring of R-C was opened by PLG–NCA, rendering emissive, ring-open R-O to initiate ROP of PLG–NCA. The emissive R-O moiety therefore produced fluorescent R–PLG with aggregation-induced emission (AIE) properties. Moreover, R–PLG was found to exhibit photochromic properties with good fatigue resistance and long lifetime when forming metal complexes with Sn(II) and Fe(III). In the dark, irradiated metal complexes slowly (~50 min) restored to the initial state. This research provides foundation for the development of new photochromic materials with long lifetime.

## 1. Introduction

Rhodamine derivatives belong to a class of the most popular luminescent dyes with long-wavelength absorption, high absorption coefficient and quantum efficiency, and good photostability in the solution state. Rhodamine dyes were therefore widely used in industrial coloration, biomarkers, molecular biology and as photosensitizers in dye-sensitized solar cells, dye laser, etc. [1,2,3,4,5,6,7,8,9]. Nevertheless, rhodamine dyes exhibit serious disadvantages, such as quenching and low photosensitivity in concentrated solution [10,11,12] or solids [13], in which rhodamine dyes packed to form non-emissive dimers [14,15]. Aggregation is therefore the key of “photo-switching” in the on–off luminescent states. With this regard, the first rhodamine-based luminogen of bisbenzofuran-bisbenzopyrano-xanthene derivative [16] with AIE [17,18,19,20,21,22,23] properties was developed since its inherent rhodamine units can be easily in the confined, aggregated state.

**Scheme 1:**
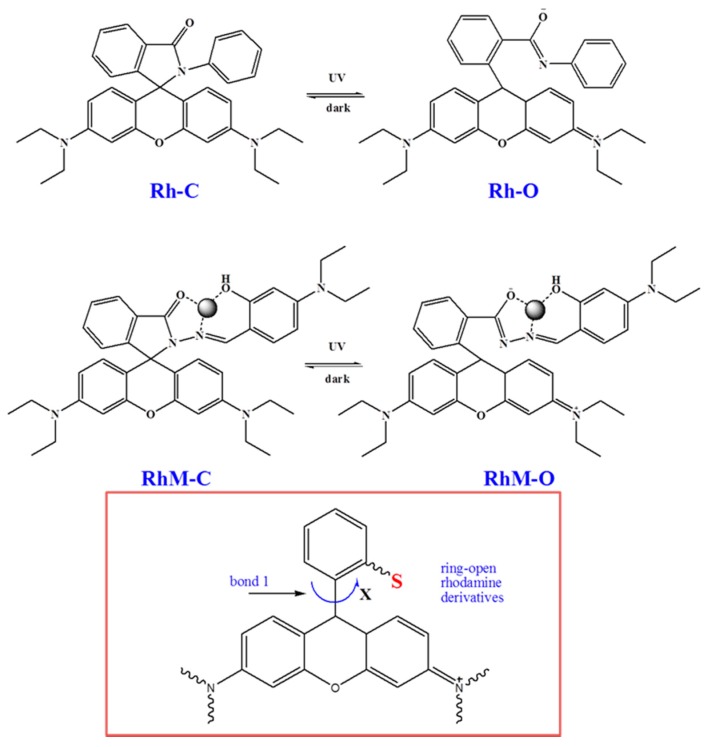
The photochromism of *N*-phenyl-rhodaminelactam (Rh–C) (**top**); rhodamine B salicylaldehyde hydrazone metal complex RhM–C (**middle**); and the rotatable bond 1 in the ring-open rhodamine derivatives (**bottom**).

In early 1977, rhodamine amide [24] had been reported to exhibit photochromic features. Later, kinetic investigation of laser light-induced reaction of *N*-phenyl-rhodaminelactam [25] (Rh–C, Scheme 1) suggested that the imidate anion (–C(–O^−^)=N–) of ring-open Rh–O is responsible for the photochromism. Unfortunately, the lifetime of the ring-open Rh–O is very short, only a few milliseconds in polar solvents. Because of short lifetime and low quantum efficiency of the photoinduced reaction, few investigations [26,27,28,29] were aimed at the photochromic properties of rhodamine amides over the next 30 years. However, recent progress suggested that metal complex (RhM–C, Scheme 1) of rhodamine B salicylaldehyde hydrazine [30] actually exhibited long lifetime and good fatigue resistance. Upon ultraviolet (UV) light irradiation, spirolactam ring of RhM–C transformed into ring-open RhM–O, which contains analogous imidate anion as binding site to result in distinct color and fluorescence changes. When the UV light was removed, the solution restored to its original state in 10 min, which suggested a long lifetime of the ring-open form.

Effective restriction [23,31,32] on the intramolecular rotation of AIE-active luminogens is the main mechanism behind AIE phenomenon. Many luminogens [17,18,19,20,21,22,23] with inherent rotational restriction have been characterized to exhibit AIE activity. We may apply the same principle to rhodamines, that is, rhodamines carrying bonds of limited rotational freedom should also be AIE-active materials. Emissive, ring-open rhodamines contain a rotatable single bond **1** (cf. bottom of Scheme 1). If rotation of bond **1** can effectively be restricted, desired AIE properties should be generated. A large, neighboring substituent **S** should serve well in hampering the rotation of bond **1**. A large polymer chain is then an ideal candidate for substituent **S** and, in our resent case, polypeptide chain was used as substituent **S** to hamper the rotation of rhodamines.

**Scheme 2:**
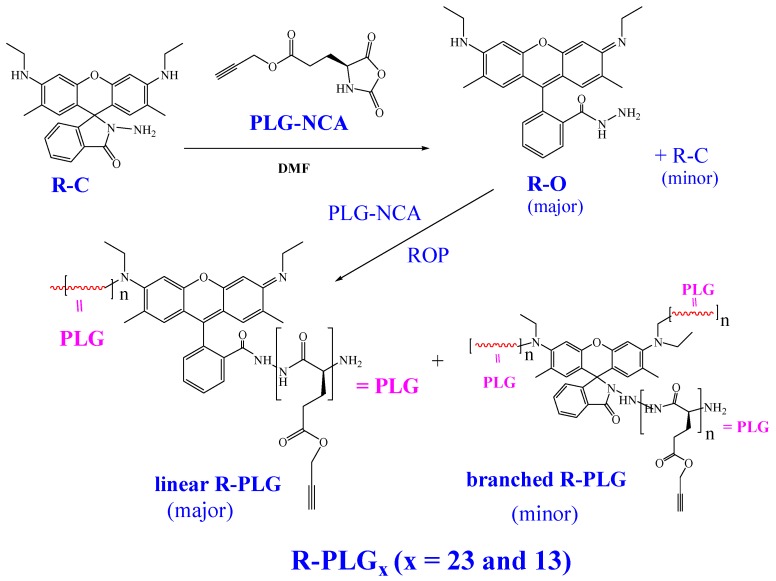
Conversion of ring-close R-C to ring-open R-O by cyclic peptide monomer PLG–NCA and the following ring-opening polymerization (ROP) of PLG–NCA by R-O initiator.

**Scheme 3:**
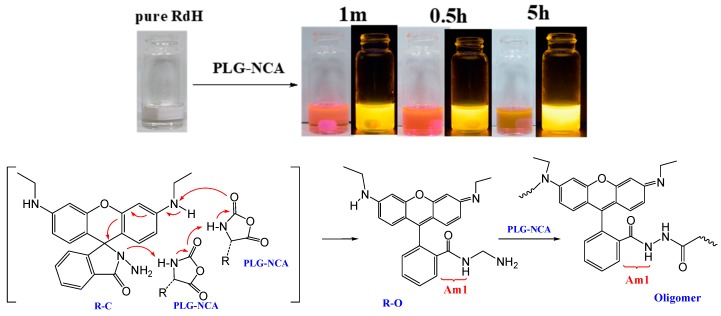
The color and luminescence changes of R-C (5 × 10^−3^ M) upon adding 100 equivalents of PLG–NCA monomer and the proposed proton-transfer process for converting ring-close R-O to R-C by PLG–NCA.

In this study, we attempted to achieve three goals. First, the role of peptide chain in promoting AIE properties of rhodamine derivative was assessed. Target rhodamine-based polypeptides of R–PLG*_x_* (*x* = 23 and 13, *x* refers to number of peptide’s repeat unit, Scheme 2) were then prepared to test this idea. These polymers were prepared by ring-opening polymerization (ROP) of PLG–NCA monomer initiated by a rhodamine hydrazide R-C. Unexpectedly and interestingly, the transparent, non-emissive solution (Scheme 3) instantaneously turned purple and emissive when PLG–NCA was added. Therefore, the role of PLG–NCA in triggering the luminescence of R-C is the second goal of this study. We then prepared R–PLG_23_ metal complexes. Similar to small-mass RhM–O [30], R–PLG_23_ metal complexes also exhibited reversible photochromism with a long lifetime of 50 min. Investigation of the photochromic properties is therefore the third goal of this study.

## 2. Results and Discussion

### Ring-Opening of R-C by the Cyclic Peptide Monomer of PLG–NCA 

Initially, we attempted to synthesize polypeptide R-PLG from ROP of PLG–NCA by R-C initiator (Scheme 1). The ^1^H NMR (Figure S1) of R-C and ^13^C NMR of R–PLG (Figure S2) demonstrate the successful preparations of initiator and polypeptide, respectively. However, despite the successful polymerization, an unexpected and interesting transformation occurred at early state of polymerization. Non-emissive, transparent R-C [33,34] (Scheme 3) became emissive and purple in color at the instant PLG–NCA [35] was added. Rhodamine is able to respond to external stimuli, such as pH, to undergo the reversible closed rhodamine spirolactam into opened amide transformation [36,37,38,39,40]. Accompanied with the structural change is the photophysical properties change; that is, the switching ON and OFF of the absorption band at about 550 nm, as well as the luminescence at about 580 nm. With the built in 2-(2-hydroxyphenyl)-benzothiazole function, rhodamine spirolactam [41] can be converted into emissive open form with a reversible excited-state intramolecular proton transfer (ESPIT) process. In contrast to ESPIT, different mechanisms should be involved in our present case. Intermolecular reaction between R-C and PLG–NCA should follow a ground-state, irreversible pathway. Presumably, the unexpected color change of the reaction mixtures is due to an intermolecular proton transfer between R-C and PLG–NCA (Scheme 3). It was envisaged that excess PLG–NCA (100 times to mole of R-C) media served as proton transfer agent, passing the proton from the ethyl amino (–Et–NH–) site to the spirolactam amide site. Accompanied with proton transfer, spirolactam of ring-close R-C was opened to result in ring-open R-O. With extended conjugation length, R-O is expected to absorb and luminesce in longer wavelength regions.

**Figure 1:**
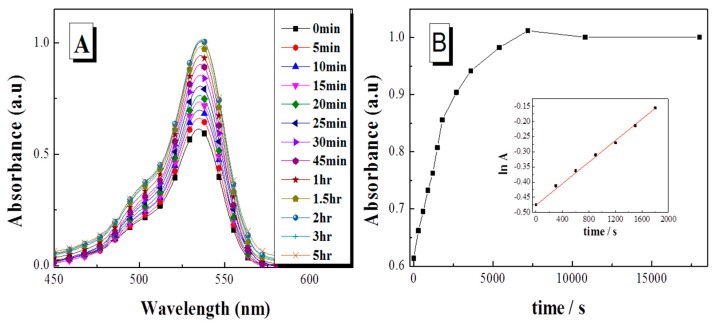
(**A**) The absorption peak of R-C/PLG–NCA mixtures ([R-C] = 5 × 10^−3^ M, [PLG–NCA] = 5 × 10^−1^ M in DMF) at 530 nm at various time after adding PLG–NCA; and (**B**) the variation of the resolved absorbance with time. Inset: The curves fitted with first-order reaction dynamics.

Pure solution of R-C in *N*,*N*-dimethyl formamide (DMF) showed no UV absorption above 500 nm but adding R-C to PLG–NCA immediately induced a new absorption peak at 530 nm (Figure 1A). This new peak is therefore associated with the ring-open R-O and, as more R-O formed over time, the new peak gradually developed its intensity and reached maximum after 2 h. Formation of ring-open R-O was characterized by the emission peak (Figure S3) at 560 nm, which also gained intensity over time. Supposedly, absorption at 530 nm is proportional to the amount of the newly-formed R-O and the ring-open kinetics of R-C can be monitored by measuring the time-dependent absorbance at 530 nm (Figure 1B). At room temperature, the resultant absorbance increased rapidly in the initial 30 min and then grew with reduced rate before reaching the ultimate plateau. Initially, excess PLG–NCA efficiently catalyzed the conversion from R-C to R-O but the depletion of PLG–NCA at later reaction step resulted in slower conversion. Within the initial 30 min when excess PLG–NCA was present in reaction mixtures, conversion from R-C to R-O solely depends on [R-C]; therefore, a *pseudo* first order kinetic was used to approach the initial conversion kinetic. Indeed, the initial absorbance curves can be fitted well with first order kinetics (inset of Figure 1B). The *pseudo* first order rate constant *k* thus evaluated is 1.77 × 10^−4^·s^−1^. In the initial 30 min when the *pseudo* first order kinetic was applied, 58 mol % of ring-close R-C had been converted to R-O. At later stage, conversion slowed down due to the depletion of PLG–NCA. However, if conversion continuously followed the fast pseudo first order reaction, 80 mol % of R-C can be converted into the desired R-O within 8000 s (~2.2 h) of reaction time. The reaction was conducted with a prolonged two days in order to ensure maximum conversion and we may conclude that the reaction products contain majority of luminescent, linear R–PLG. Non-emissive branched R–PLG (cf. Scheme 2) would be present as minor product in the polymerized mixtures.

**Figure 2:**
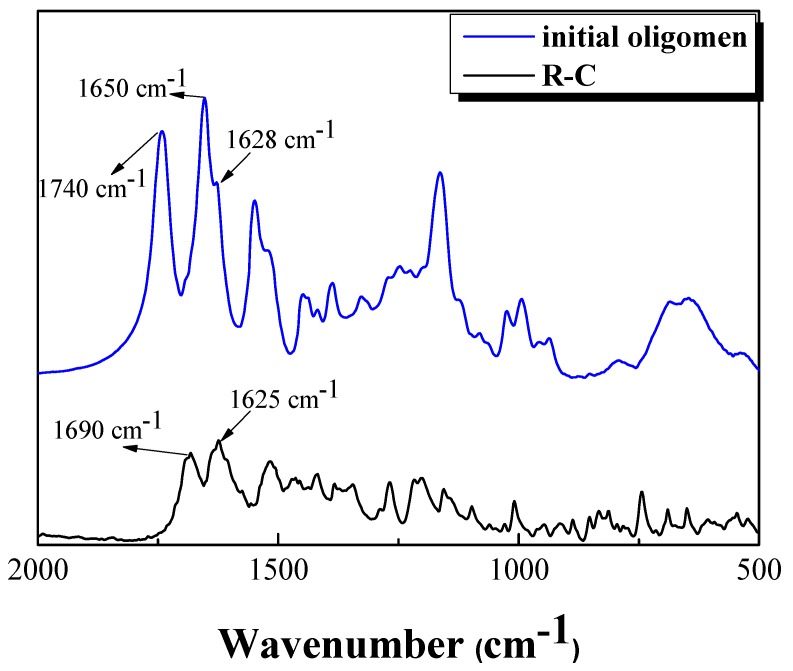
Solid FTIR spectra of pure R-C and the quenched oligomer from the R-C/PLG–NCA mixtures.

It will be more persuasive and meaningful if we can spectrally identify the ring-open product formed at the early state of reaction. Quickly after mixing R-C and PLG–NCA in tetrahydrofuran (THF), we performed precipitation of the solution mixtures from diethyl ether to obtain the early-stage product (presumably, peptide oligomers with high content of ring-open R-O moiety). According to Scheme 3, R-O moiety in the quenched oligomer contains an amide group (Am1), which absorbs and resonates differently from the rest of amide groups in the peptide chain. Am1 group of R-O moiety can be spectrally distinguished from the rest amide groups in the peptide chain. Fourier-transform infrared spectroscopy (FTIR) spectra of R-C and the quenched oligomer were then compared in Figure 2. Amide group of the spirolactam in R-C absorbs at 1690 and 1625 cm^−1^. A quick comparison indicated that the large amide absorption at 1690 cm^−1^ is no longer seen in the spectrum of quenched oligomer. Instead, amide groups of oligomer absorb at positions (1650 and 1628 cm^−1^) different from those of R-C. For R-C, the amide absorption bands resembled those in the spectrum of polymeric R–PLG*_x_* (as will be discussed in next section), which includes the free C=O of the peptide side chains at 1740 cm^−1^ [42]. Most of the R-C molecules can be converted into R-O in THF solvent regarding the good proton transfer capability of THF. Suggestively, DMF is inferior to THF in proton transfer but only DMF can be used as polymerization solvent for its capability to dissolve the polypeptide products.

## 3. Characterizations of R–PLG*_x_*

As illustrated in Scheme 2, the target polymer R–PLG*_x_* was prepared from ROP of PLG–NCA initiated by R-C. Because of the incomplete conversion from R-C to R-O, we assumed that the resultant R–PLG*_x_* contained majority of linear R-PLG besides minor, branched R–PLG. The peptide monomer PLG–NCA [42,43] was prepared from the facile cyclization of propargyl-glutamate by triphosgene. Amino-functionalized rhodamine initiator R-C was prepared from reaction of rhodamine B and hydrazine according to the reported procedures [44,45]. By adjusting the feeding ratio between NLG–NCA and R-C, two polypeptides R–PLG_13_ and R–PLG_23_ (the subscript refers to number of repeat unit) were prepared. Detailed synthesis procedures for all intermediates and products are described in the Supplementary Materials and spectral identifications of the peptide products are given and discussed below.

**Figure 3:**
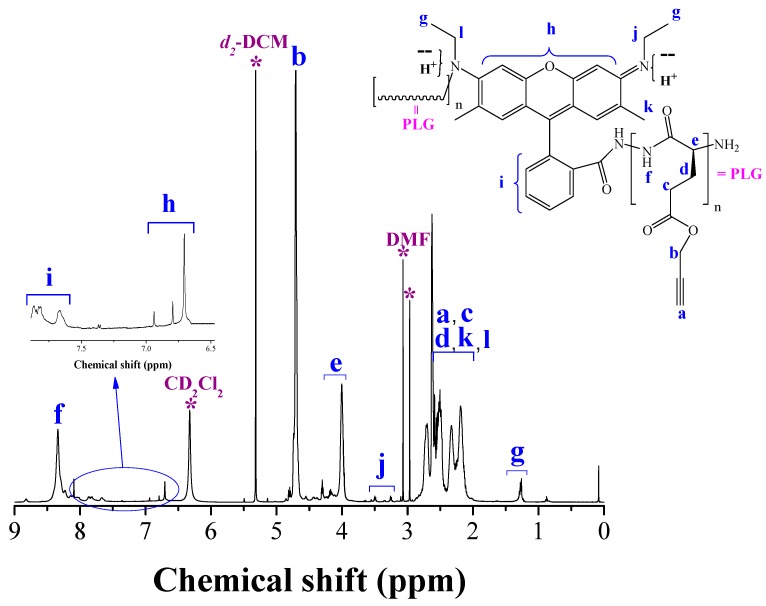
^1^H NMR spectrum of R–PLG_23_ in CD_2_Cl_2_ /TFA (trace).

For proton nuclear magnetic resonance (NMR) analysis of R–PLG_23_ in CD_2_Cl_2_ (Figure 3), a small amount of trifluoroacetic acid was added to enhance dissolution of the rigid polypeptide. Protonated –EtNH^+^ and –C=NH^+^ thereby lowered the electron density of the neighboring xanthene ring, rendering two small resonance peaks of *H*_h_ (cf. the magnified peaks) in the down-field region. Integration ratio between the whole xanthene protons *H*_h_ and main-chain protons *H*_e_ was used to evaluate *M*_n_ (Table 1). The resultant *M*_n_ reasonably correlates with the result from matrix-assisted laser desorption/ionization-time of flight mass spectroscopy (Figure S4). In Figure 3, only resonance peaks from the linear chain polypeptide was observed, which may correlate with the hypothesis that linear R-PLG_23_ is the major product from the polymerization run. Side-chain protons *H*_a_, *H*_c_ and *H*_d_ resonate as overlapped multiplets in the range from 2.17 to 2.73 ppm. In contrast, resonances of protons *H*_b_ and *H*_e_ in the side- and main-chains are distinct singlets at 4.70 and 3.98 ppm, respectively.

**Figure 4:**
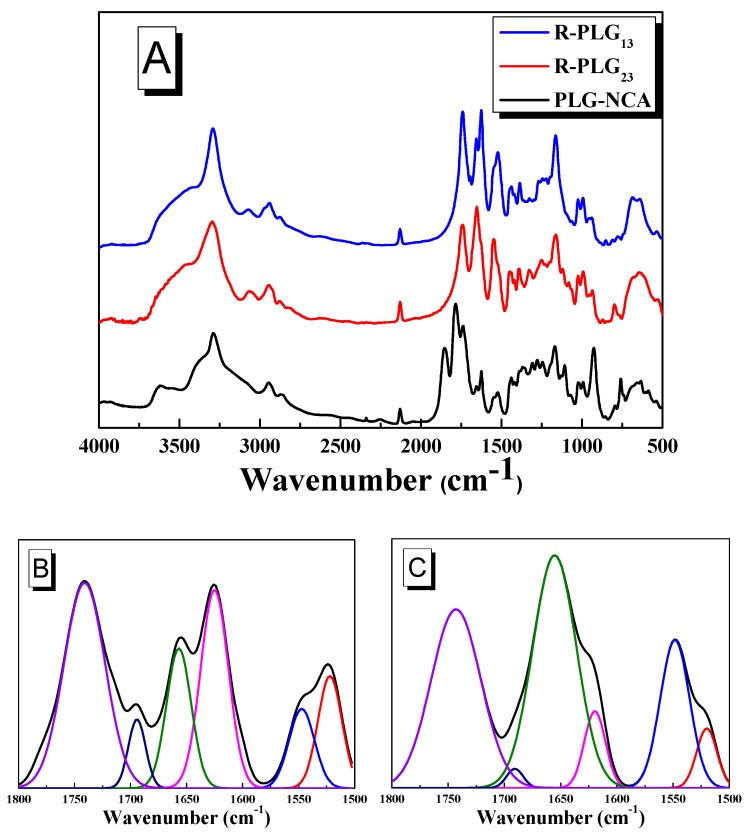
Solid FTIIR spectra of: (**A**) R-C, R–PLG_13_ and R–PLG_23_; and the de-convoluted amide I absorption peaks of: (**B**) R–PLG_13_; and (**C**) R–PLG_23_.

FTIR analysis (Figure 4A) can be used to demonstrate the success of polymerization since the spectra of polypeptides contain no anhydride carbonyl stretching of the starting PLG–NCA at 1849 cm^−1^. Previously, analysis of FTIR spectra using the second-derivative technique [42] revealed that the amide I band at 1655 cm^−1^ is characteristic of the α-helical chain and the amide I band at 1627 cm^−1^ is typical of β-sheet conformation while for turn populations, a characteristic peak at 1693 cm^−1^ should be resolved. Moreover, the free C=O group of the peptide side chains provides a signal at 1740 cm^−1^. The absorption peaks in the range from 1500 to 1800 cm^−1^ were then de-convoluted (Figure 4B,C) for determining fraction of each secondary structure. The results summarized in Table 1 clearly indicate that high molecular weight (*M*_W_) R–PLG_23_ is higher in content of α-helical chains compared to low *M*_W_ R–PLG_13_. For polypeptides, α-helix can be regarded as rigid rods stabilized through intramolecular hydrogen bonds (H bonds), whereas β-sheets are structures stabilized by intermolecular H bonds [44,45]. Previous report [46] also concluded that high *M*_W_ polypeptides always contain more fraction of rigid α-helix chain. Suggestively, long peptide chains are more efficient in sterically shielding the main chain amides from intermolecularly H bonding to others. Therefore, α-helical structure with predominant intramolecular H bonds preferably formed in high *M*_W_ polypeptide. As will be discussed later, high fraction of α-helical chain for R–PLG_23_ also affected its emission behavior.

Circular dichroism (CD) was applied to evaluate fraction of secondary structures of R–PLG*_x_* in methanol. A Jasco J-810-150S spectropolarimeter (JASCO International CO. Ltd., Tokyo, Japan) equipped with Jascow32 Spectral Manager program (JASCO International CO. Ltd., Tokyo, Japan) were used for CD measurement and curve-fitting, respectively. Corresponding CD spectra (Figure S5) were then fitted to resolve the percentages of each secondary structure and the results are summarized in Table 1 to compare with those obtained from FTIR. It is clear that polypeptides in methanol are much lower in α-helical chains compared to polypeptide in solid state. Presumably, methanol media tend to intermolecularly H bond to amide groups of the α-helical chains, lowering α-helical chains stabilized by intramolecular H bonds. Despite the difference in the resolved values, the CD result still suggests that high *M*_W_ R–PLG_23_ has higher fraction of α-helical chains than the low *M*_W_ R–PLG_13_.

## 4. Emissive Properties of R–PLG_13_ and R–PLG_23_

Small-mass rhodamine 6G is a luminogen exhibiting aggregation-caused quenching [43,44,45] properties. Dilute solution of rhodamine 6G emits with higher emission efficiency than its concentrated solution state. In this study, we nevertheless found that incorporation of peptide chains in R–PLG*_x_* reversely transformed inherent rhodamine R-O moiety into AIE-active luminogen. The AIE properties of R–PLG*_x_* were evaluated according to its emission behavior toward aggregation and concentration.

**Figure 5:**
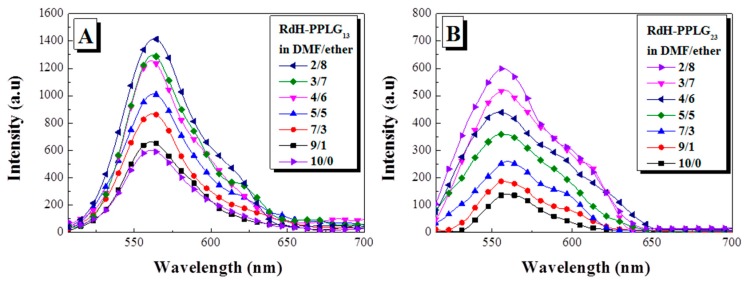
Emission spectra of: (**A**) R–PLG_23_ (10^−4^ M); and (**B**) R–PLG_13_ (10^−4^ M) in solution mixtures of DMF/ether of different compositions (λ_ex_ = 490 nm).

Because AIE activity can be promoted by aggregation, diethyl ether was employed as poor solvent to induce aggregation of R–PLG*_x_* in a good solvent of DMF. Upon irradiation at 490 nm, pure solution of R–PLG_23_ (10^−4^ M) in DMF already emitted with discernible intensity (Figure 5A) but the inclusion of diethyl ether (while keeping concentration of R–PLG_23_ constant at 10^−4^ M) further raised the solution emission intensity. Solution of R–PLG_13_ (10^−4^ M) behaved similarly (Figure 5B) but with comparatively lower emission intensity than R–PLG_23_. The enhanced emission induced by diethyl ether is correlated with the size of aggregated particles, which can be monitored by dynamic light scattering (DLS). Results (Figure S6) from DLS indicate that the aggregated particles continuously shrunk in size as more diethyl ether was added in the solution. Suggestively, rotation of the luminescent R-O moiety in R–PLG*_x_* is more effectively restricted if the R-O moiety was confined in the limited spaces of the shrunken particles. The hampered molecular rotations in the shrunken nanoparticles therefore promote the AIE-related emission by blocking the non-radiative decay pathways coupled with molecular rotation. Therefore, addition of poor solvent should enhance emission of R-O moiety in R–PLG*_x_*.

We also identified AIE properties by concentration effect. By increasing the solution concentration of R–PLG from 10^−4^ to 10^−2^ M, we observed more than 50-fold intensity enhancement (Figure S7) for both systems of R–PLG_23_ and R–PLG_13_. Higher aggregation level in more concentrated solution is responsible for the emission enhancement here.

The solution quantum efficiency (Φ_F_) of R–PLG_23_ and R–PLG_13_ is summarized in Table 2. The resultant Φ_F_ suggests that R–PLG_23_ solutions are always higher in emission efficiency than R–PLG_13_ solutions under the same experimental condition. Theoretically, low *M*_W_ R–PLG_13_ should be higher in luminescent R-O content compared to high *M*_W_ R–PLG_23_. However, high R-O content in R–PLG_13_ does not benefit emission efficiency. Therefore, another decisive factor is involved here. Luminescent R-O is suggested to be hampered in rotation if it was linked by rigid α-helical chain. R–PLG_23_ contains higher α-helical chain and its R-O moiety should be more hampered in rotation compared to R-O in R–PLG_13_. Since RIR [23,31,32] is the mechanism responsible for AIE, R–PLG_23_ with high content of α-helical chains should be more efficient in emission compared to R–PLG_13_.

## 5. Metal Chelation 

**Scheme 4:**
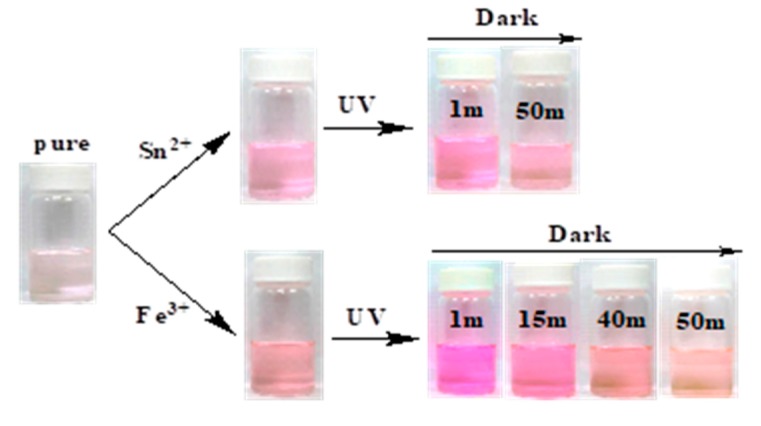

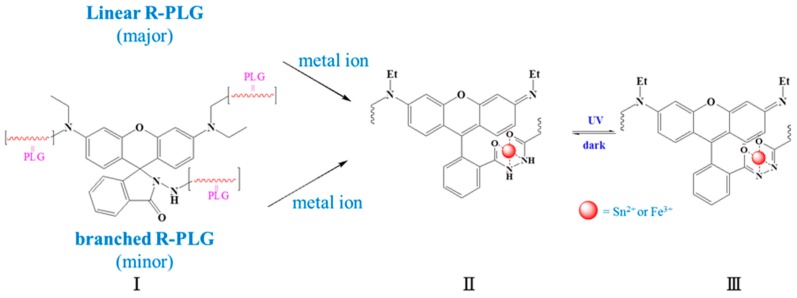
The color and luminescence changes of R–PLG_23_ (10^−4^ M) metal complex solutions in THF upon adding metal ions (10^−3^ M) and UV irradiation (**top**); and the proposed mechanism for the photochromism of metal complexes (**bottom**).

**Figure 6:**
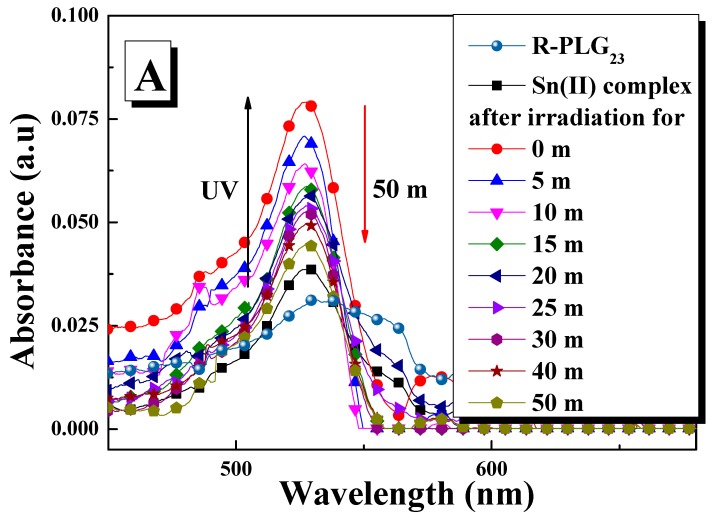

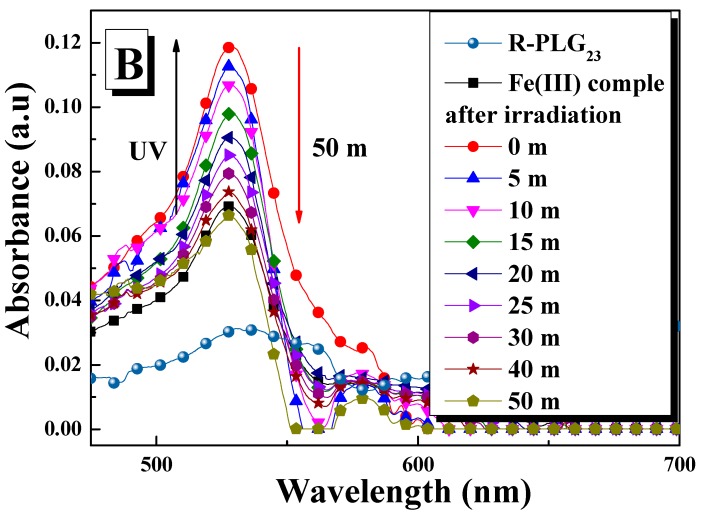
Solution UV absorbance spectra of: (**A**) Sn(II); and (**B**) Fe(III) R–PLG_23_ metal complexes after irradiation for different time ([SnCl_2_] = 10^−3^ M, [FeCl_3_] = 10^−3^ M and [R–PLG_23_] = 10^−4^ M in THF).

To afford sufficient metal complexes of R–PLG_23_, 10-fold Sn(II) or Fe(III) was added to solution of R–PLG_23_ in THF. As illustrated in Scheme 4, light-purple solution became deeper in color when Sn(II) was added. Upon UV irradiation, color of the solution became deeper and deeper over time. The deepening color over time can be evaluated by time-dependent UV absorption spectra, which will be discussed later (Figure 6). When UV light was removed, the solution gradually restored to its original state, with the same absorption intensity before UV irradiation, in more than 50 min. The metal complexes of R–PLG_23_ are therefore photoreversible materials with extremely long lifetime. The UV light-induced color change was also characterized by the enhanced emission at 560 nm (Figures S8 and S9).

Metal complexation slightly enhanced the absorption peak of R–PLG_23_ (Figure 6) at 525 nm but further UV irradiation immediately and largely raised this absorption peak. When the UV light was removed, the irradiated solution was kept in the dark for varied times before UV analysis. In the dark, absorption peak of the irradiated solution gradually reduced its intensity over time but, after the UV light had been removed for 50 min, the detected absorbance is still slightly higher than the initial value before irradiation. The metal complexes of R–PLG_23_ are therefore photochromic systems with extremely long lifetime (>50 min).

**Figure 7:**
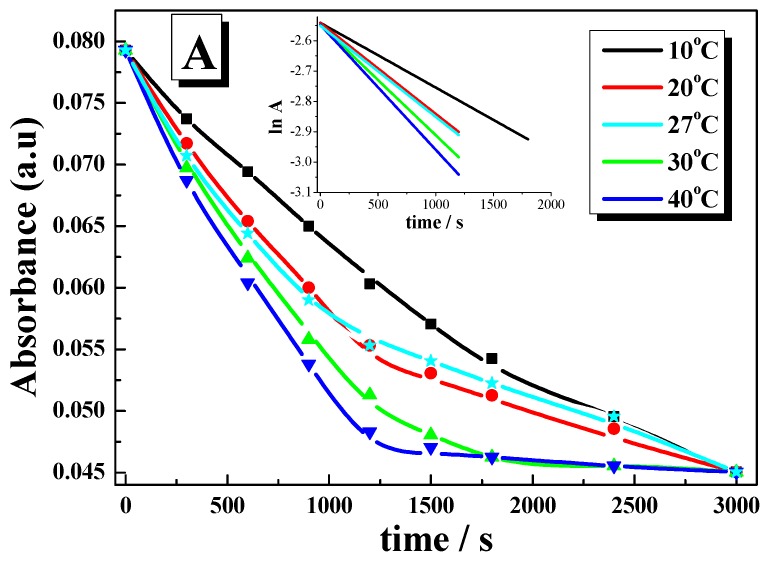

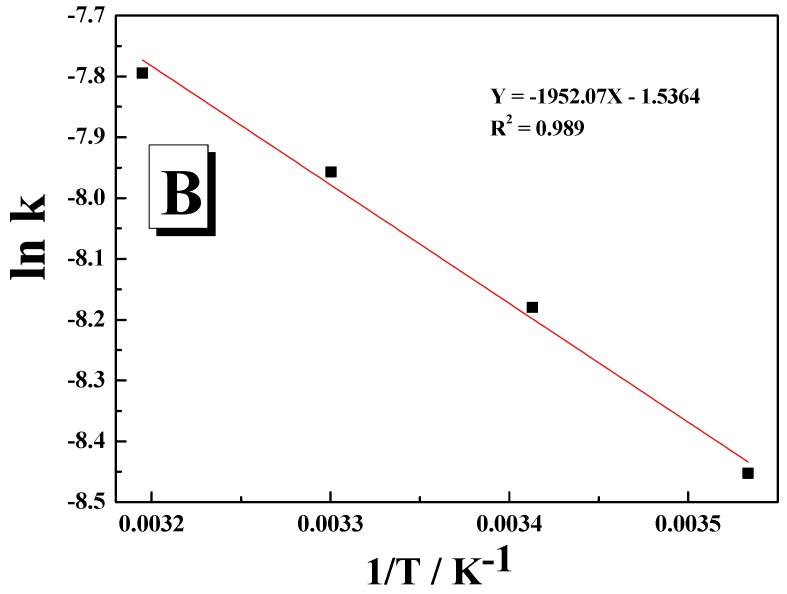
The thermal fading kinetics of: (**A**) Sn(II)-chelated R–PLG_23_ metal complexes after UV irradiation for different temperatures ([SnCl_2_] = 10^−3^ M, and [R–PLG_23_] = 10^−4^ M in THF). Inset: The curves fitted with first-order reaction dynamics ln*A* = −*kt* to calculate the thermal bleaching speed constant: *k* = 2.13 × 10^−4^ s^−1^ (10 °C), *k* = 2.99 × 10^−4^ s^−1^ (20 °C), *k* = 3.01 × 10^−4^ s^−1^ (27 °C), *k* = 3.64 × 10^−4^ s^−1^ (30 °C), *k* = 4.12 × 10^−4^ s^−1^ (40 °C). (**B**) The data were fitted with Arrhenius expressions.

**Figure 8:**
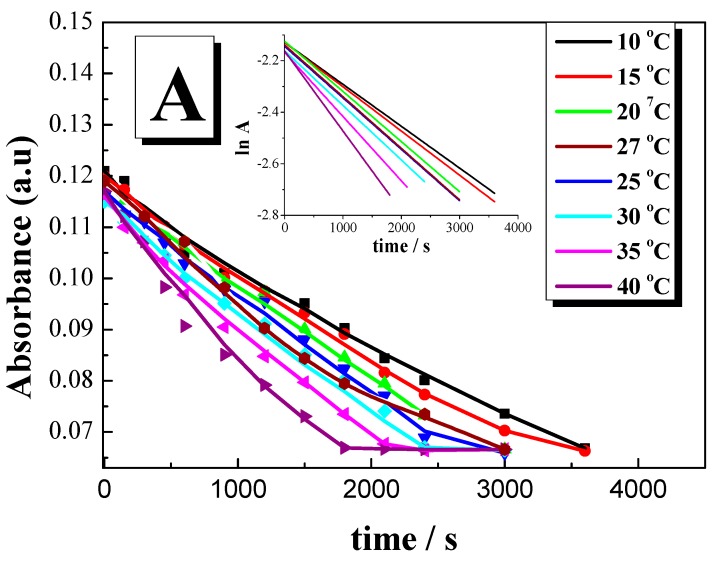

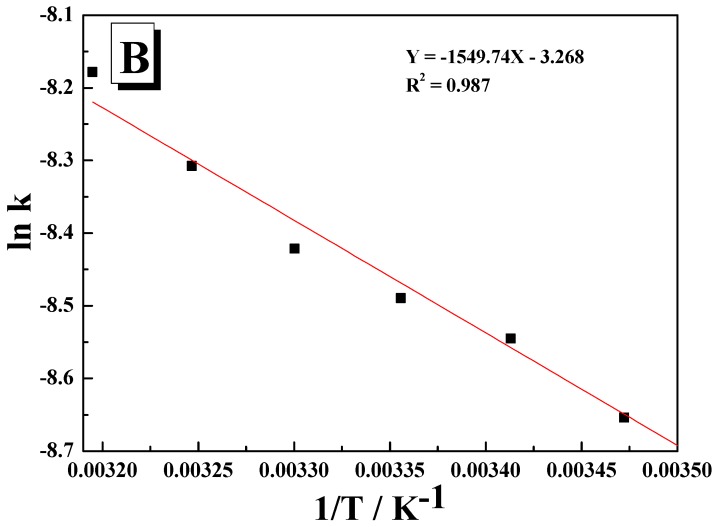
The thermal fading kinetics of: (**A**) Fe(III)-chelated R–PLG_23_ metal complexes after UV irradiation for different temperatures ([FeCl_3_] = 10^−3^ M and [R–PLG_23_] = 10^−4^ M in THF). Inset: The curves fitted with first-order reaction dynamics ln*A*= −*kt* to calculate the thermal bleaching speed constant: *k* = 1.62 × 10^−4^ s^−1^ (10 °C), *k* = 1.74 × 10^−4^ s^−1^ (10 °C), *k* = 1.94 × 10^−4^ s^−1^ (20 °C), *k* = 2.06 × 10^−4^ s^−1^ (25 °C), *k* = 2.22 × 10^−4^ s^−1^ (30 °C), *k* = 2.47 × 10^−4^ s^−1^ (20 °C), *k* = 2.81 × 10^−4^ s^−1^ (40 °C). (**B**) The data were fitted with Arrhenius expressions.

Thermal fading rates of photo-excited Sn(II)- and Fe(III)-R–PLG_23_ were also evaluated from the absorbance changes (Figure 7A and Figure 8A) at temperatures from 10 to 40 °C. Primarily, the logarithmic absorbance (ln*A*) at different temperatures can be linearly correlated with the fading time (insets of Figure 7A and Figure 8A). Using the Arrhenius expression (ln*k* = −*E*_a_/*RT* + constant), the rate constants (*k*) could be evaluated. The activation energies for the recovery of photo-excited Sn(II)- and Fe(III)-R–PLG_23_ were then evaluated from the linear plots of ln(*k*) versus 1/*T* (Figure 7B and Figure 8B) and are 16.24 and 12.88 kJ/mol, respectively. This result suggests the lifetime of Sn(II)- and Fe(III)-R–PLG_23_ could be increased by lowering the temperature.

Hypothetically, hydrazide groups of the major, linear polypeptide (Scheme 4) and spirolactam ring of the minor, branched polypeptide I are potential chelating sites for metal ions. Primarily, metal ions acted to convert the spirolactam ring of the branched polypeptide I into metal ion-chelated amide unit in the ring-open species II. Because content of polypeptide I is low, absorption enhancement upon metal ion chelation is therefore also low. Major absorbance jump I is due to UV irradiation, which transformed most of the hydrazide groups in species II into two imidate anions in specie III. This structural transformation may proceed readily because the absorption enhancement occurred exclusively. In the dark, the ring-open species III slowly transformed back into the initial species II and this process was characterized by a long lifetime of >50 min.

## 6. Conclusions

Two polypeptides of R–PLG_13_ and R–PLG_23_ with luminescent R-O moiety were prepared from ROP of cyclic PLG–NCA monomer initiated by a non-emissive R-C. At early stage of ROP, R-C was converted by PLG–NCA, rendering emissive R-O as the major initiator for the following polymerization.

In contrast to the general concept that small-mass rhodamine derivatives are luminogens with traditional aggregation-caused quenching properties, the resultant polypeptides are nevertheless AIE-active luminogens. The polypeptide chains imposed effective rotational restriction on inherent R-O moiety to result in AIE activity of R–PLG*_x_*. The high *M*_W_ R–PLG_23_ is higher in α-helix content, thereby, it is also a more efficient luminogen compared to low *M*_W_ R–PLG_13_ with lower α-helix content. The roles of secondary structure on restricting rotation motion and on the AIE-related emission are therefore clarified.

Upon metal chelation, R–PLG_23_ exhibited slight enhancement on the absorption and emission peaks. Nevertheless, further UV irradiation on the metal complexes largely promoted the absorption and the luminescence, and the photo-excited metal complexes underwent slow recovery with a lifetime >50 min. The polypeptide metal complexes examined in this study are therefore new members of the photochromic family with advantages such as long-wavelength absorption and long lifetime.

## Figures and Tables

**Table 1:** Molecular weight of R–PLG_13_ and R–PLG_23_ evaluated from ^1^H NMR and Maldi-Tof mass spectra and fractions of secondary structure determined from FTIR and CD spectra.

Sample	*M*_W_ (Dalton/mol)			Secondary structure (%)			
	*M*_n,NMR_^a^	*M*_n,mass_^b^	*M*_w,mass_^b^	a-Helix	b-Sheet	Turn	Random coil
R–PLG_13_	1837 ^a^	2147 ^b^	2323 ^b^	36.3 ^c^(13.6) ^d^	52.4 ^c^(38.6) ^d^	11.3 ^c^(22.5) ^d^	-(25.4) ^d^
R–PLG_23_	3871 ^a^	3815 ^b^	4396 ^b^	83.7 ^c^(49.5) ^d^	13.4 ^c^(28.3) ^d^	2.9 ^c^(16.6) ^d^	-(5.5) ^d^

^a^ Determined by the integral ratio between *H*_h_ and *H*_e_ in ^1^H NMR spectrum in Figure 3; ^b^ Determined by Maldi-Tof mass spectra in Figure S4; ^c^ Fraction of the resolved area (*A*_f_, %) from the de-convoluted infrared spectra in Figure 4B,C; ^d^ Fraction of secondary structure calculated from the CD spectra in Figure S5.

**Table 2:** Quantum efficiency (Φ_F_, %) of R–PLG_23_ and R–PLG_13_ in solution mixtures of DMF/ether and in DMF of different concentrations.

Sample	Φ_F_ (%) of R–PLG in DMF/ether ^a^ with volumetric ratio of *x*/*y* =							Φ_F_ (%) of R–PLG in DMF with concentration of		
	10/0	9/1	7/3	5/5	4/6	3/7	2/8	10^−4^ M	10^−3^ M	10^−2^ M
R–PLG_23_	1.1	1.4	1.9	3.1	4.2	5.0	6.1	1.1	13.8	62.5
R–PLG_13_	0.8	0.9	1.1	1.4	1.6	1.7	1.9	0.8	2.7	46.7

^a^ Polymer concentration in the solution mixtures is kept constant at 10^−4^ M.

## Supplementary Materials

Supplementary Materials are available online at www.mdpi.com/2073-4360/9/9/419/s1.

## Abbreviated Terminology

ROP	ring-opening polymerization
PLG–NCA	γ-propargyl-l-glutamate *N*-carboxyanhydride
R-C	rhodamine 6G hydrazide
AIE	aggregation-induced emission
Rh–C	*N*-phenyl-rhodaminelactam
UV	ultraviolet
ESIPT	excited-state intramolecular proton transfer
DMF	*N*,*N*-dimethyl formamide
THF	tetrahydrofuran
FTIR	Fourier-transform infrared spectroscopy
NMR	nuclear magnetic resonance
*M*_W_	molecular weight
CD	circular dichroism
DLS	dynamic light scattering
