# Supplementary material for: Light-Up of Rhodamine Hydrazide to Generate Emissive Initiator for Polymerization and to Afford Photochromic Polypeptide Metal Complex
Source: Polymers (Basel). 2017 Sep 5;9(9):419. doi: 10.3390/polym9090419 (PMC6418603; doi:10.3390/polym9090419)
Supplement: Supplementary file 1 [file polymers-09-00419-s001.pdf]

Light-up of rhodamine hydrazide to generate emissive initiator for polymerization and to afford photochromic polypeptide metal complex

Jhen-Yan Gao, Wen-Chih Huang, Pei-Yi Huang, Cheng-Yu Song and Jin-Long Hong*

Department of Materials and Optoelectronic Science, National Sun Yat-Sen University, Kaohsiung 80424, Taiwan

Experimental section

Materials

DMF (Aldrich) was refluxed and distilled over CaH_2 (Aldrich) under reduced pressure to a flask containing alumina before use. Rhodamine 6G (Acros, 99%) and hydrazine monohydrate (Aldrich, 80%) were used without further purification. γ-Propargyl-L-glutamate N-carboxyanhydride (PLG-NCA) were prepared according to the reported procedures [s1]. Preparation procedures of R-C and R-PLG were illustrated in Scheme S1 and detailed as below:

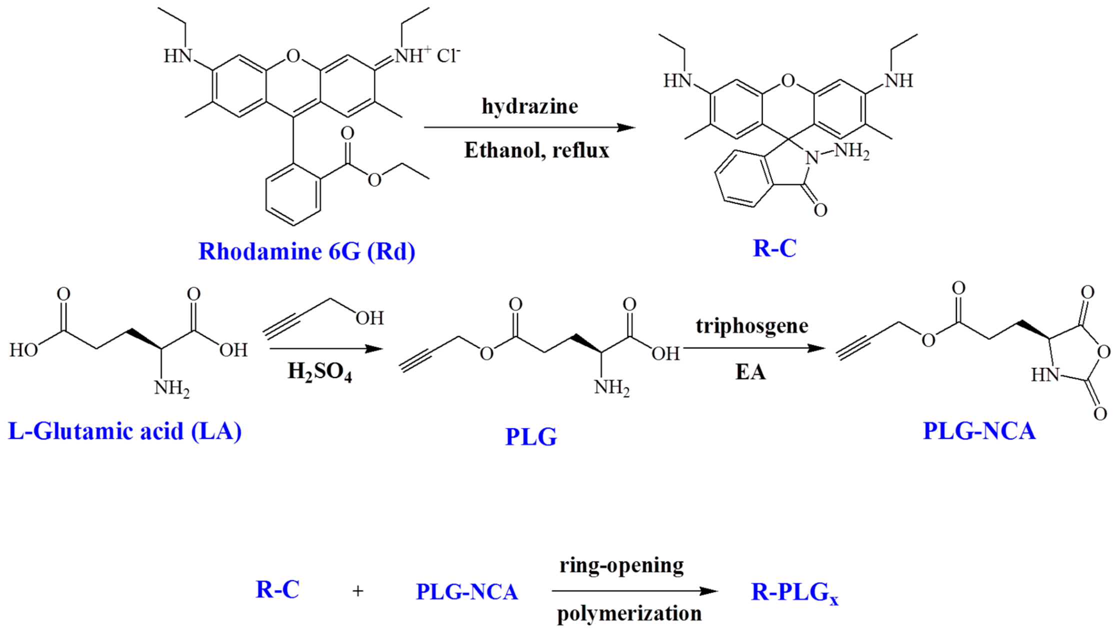

Scheme S1. Syntheses of R-C and PLG-NCA and the following ring-opening polymerization of PLG-NCA monomer initiated by R-C to obtain R-PLG_x.

Preparations of Rhodamine hydrazide (R-C).

R-C was synthesized by using the method previously described [s2,s3]. To a vigorously-stirred solution of rhodamine 6G (2 g, 4 mmol) in ethanol (65 mL), hydrazine monohydrate (6.3 mL, 110 mmol) was added dropwise at room temperature. The mixture was then heated under reflux for 2 h. After cooling to room temperature, the mixture solution was filtered and the solid filtrate was thoroughly washed with ethanol–water (1 : 2) to provide R-C as a pink solid (yield 1.51 g, 87%). ^{1}H NMR (300 MHz; d_6-DMSO): δ 1.21 (6H, t, CH_3), 1.87 (6H, s, CH_2CH_3), 3.10–3.16 (4H, m, NCH_2), 4.21 (2H, s, NH_2), 4.99 (2H, t, J = 5.44 Hz, NH), 6.10 (2H, s, ArH), 6.27 (2H, s, ArH), 6.92–6.94 (1H, m, CH), 7.44–7.48 (2H, m, ArH), 7.74–7.77 (1H, m, ArH). (Figure S1)

Ring-opening polymerization of N-carboxyanhydride to prepare R-PLG$_{13}$ and R-PLG$_{23}$

Solution of PLG-NCA (3 g, 14.2 mmol) in anhydrous DMF (20 mL) was stirred and bubbled with argon for 20 min before adding solution of R-C (0.20 g (0.47 mmol) for R-PLG$_{13}$ and 0.086 g (0.2 mmol) for R-PLG$_{23}$ in anhydrous DMF (5 mL). The reaction mixture was stirred for 2 days at room temperature and the resulting polymer was precipitated from diethyl ether, and dried in vacuum oven. ^{1}H NMR (500 MHz, CD_2Cl_2): δ 8.34 (broad, 1H, H_f), 6.1-7.63 (broad, 8H, H_h, H_i), 4.68 (s, 2H, H_b), 3.98 (broad, 1H, H_e), 3.5, 3.2 (2H, H_j), 2.72 (broad, 2H, H_c), 2 - 2.8 (m, $H_{a,c,d,k,l}$), 1,25 (4H, H_g) (Figure 3). ^{13}CNMR (125 MHz, CD_2Cl_2): δ 177.2 ($C_{f'}$), 173.0 ($C_{h'}$), 163 ($C_{6'}$), 132-128 ($C_{5'}$), 96-94 ($C_{4'}$), 79.3 ($C_{g'}$), 76.0 ($C_{a'}$), 57.5 ($C_{b'}$), 52.9 ($C_{e'}$), 41.0 ($C_{3'}$), 31.9 ($C_{c'}$), 26.4 ($C_{d'}$)., 19.0 ($C_{2'}$), 16.0 ($C_{1'}$) (Figure S2).

Measurements

^{1}H NMR spectra were recorded at room temperature using a Bruker AM 500 (500 MHz) spectrometer with tetramethylsilane (TMS) as external standard. To ensure thorough dissolution of the polymers to afford clearly-resolved resonance peaks, one drop of trifluoroacetic acid (TFA) was added to sample solution in CD_2Cl_2. ^{13}C CP-MAS NMR spectra were acquired on a Bruker 14.1 T wide-bore Avance III spectrometer equipped with a 4 mm double-resonance magic-angle-spinning (MAS) probe head. The Larmor frequency used for ^{13}C NMR was 150.92 MHz and the samples were spun at 12 kHz. The molecular weights (MW) of R-PLG$_{23}$ and R-PLG$_{13}$ were determined by a Bruker Autoflex III Maldi-Tof mass spectrometer. The *trans*-2-[3-(4-*tert*-Butylphenyl)-2-methyl-2-propenylidene]malononitrile (DCTB) containing 1 wt% sodium and potassium salts was used as matrix material. Sample was mixed with the matrix in a weight ratio of 1/10 in acetonitrile to induce crystalline material for analysis. The sample spot was irradiated with a Nd: YAG laser (355 nm; pulse duration, 3 ns; 200 Hz) for desorption and ionization. In total, 1400 laser shots are averaged for each sample analysis to obtain representative mass spectra. Infrared spectra were recorded using a Bruker Tensor 27 Fourier-transform infrared (FTIR) spectrophotometer; 32 scans were collected at a spectral resolution of 1 cm^{-1}. The solid polymer powders were homogeneously blended with KBr before being pressed to make pellets for measurements. The UV absorption spectra were recorded with a JASCO V-770 spectrophotometer. The PL emission spectra were obtained from a LabGuide X350 fluorescence spectrophotometer using a 450 W Xe lamp as the continuous light source. A small quartz cell with dimensions $0.2 \times 1.0 \times 4.5$ cm^3 was used to accommodate the solution sample. A "right angle" geometry was employed such that fluorescence was collected at a right angle to the excitation beam used in the spectral measurement. Circular dichroism (CD) spectra were recorded using a JASCO J-810-150S (Japan) spectropolarimeter equipped with a Jascow32 Spectral Manager program. Polymer

solutions with constant concentration of 10^{-4} M were used for measurement. The spectra were also curve-fitted using the Jascow32 Spectra Manager program to resolve the fraction of the individual secondary structure. The dimension of the aggregate particles was determined using a dynamic light scattering (DLS) instrument on a Malvern ZetaSizer Nano ZS90 spectrometer. A He –Ne laser operating at 633 nm was used as the light source.

References

[s1] Y. C. Lin and S. W. Kuo, *Polym. Chem.* **2012**, 3, 162.

[s2] S. Kang, S. Kim, Y. K. Yang, S. Bae, J. Tae, *Tetrahedron Letters* **2009,** 50, 2010.

[s3] J. Madsen, N. J. Warren, S. P. Ames. *Biomaterials* **2011,** 12, 2225.

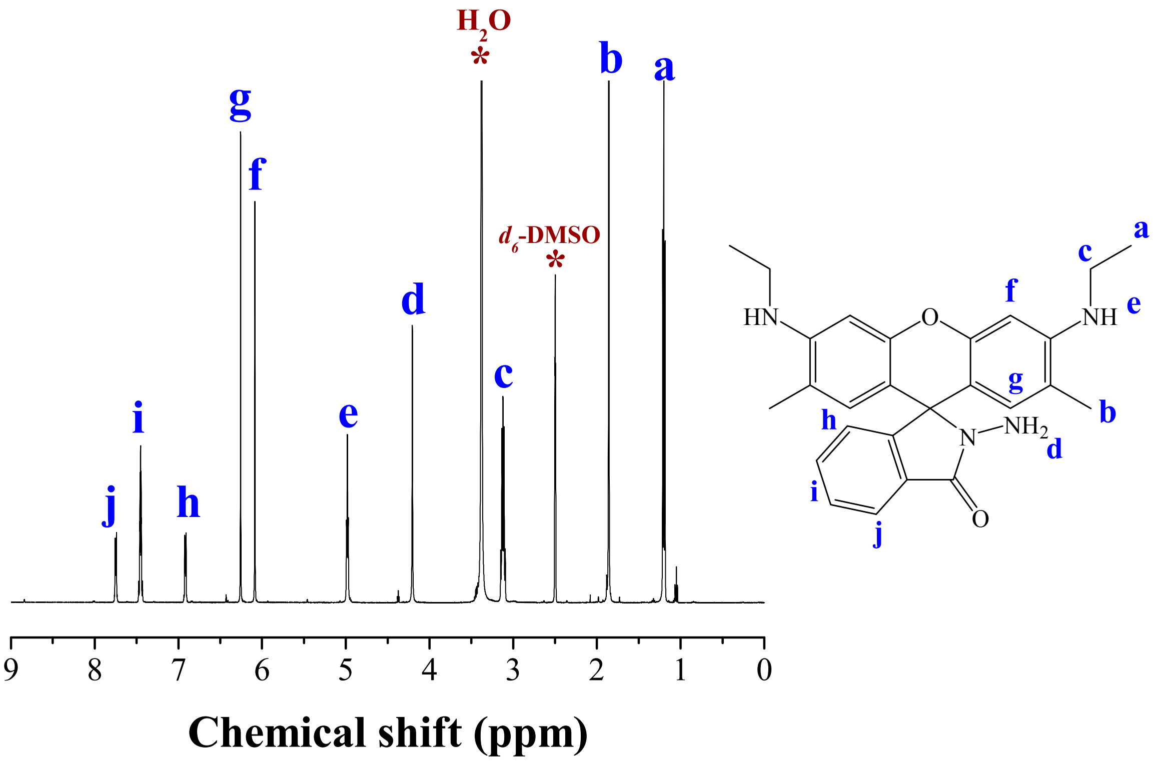

Figure S1. ^{1}H NMR spectra of R-C (in d_6-DMSO).

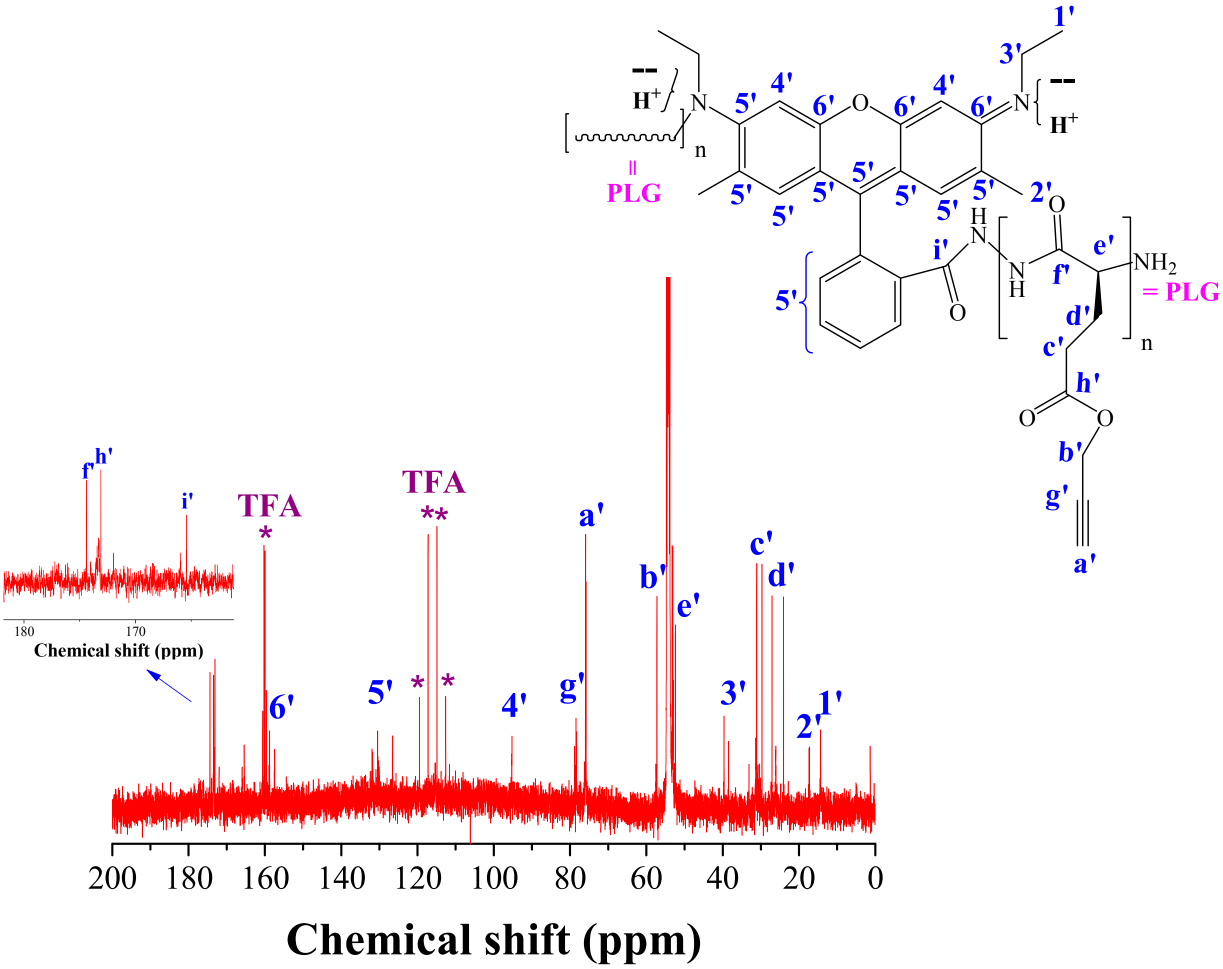

Figure S2 ^{13}C NMR spectra of R-PPLG$_{13}$ in CD_2Cl_2/TFA(trace).

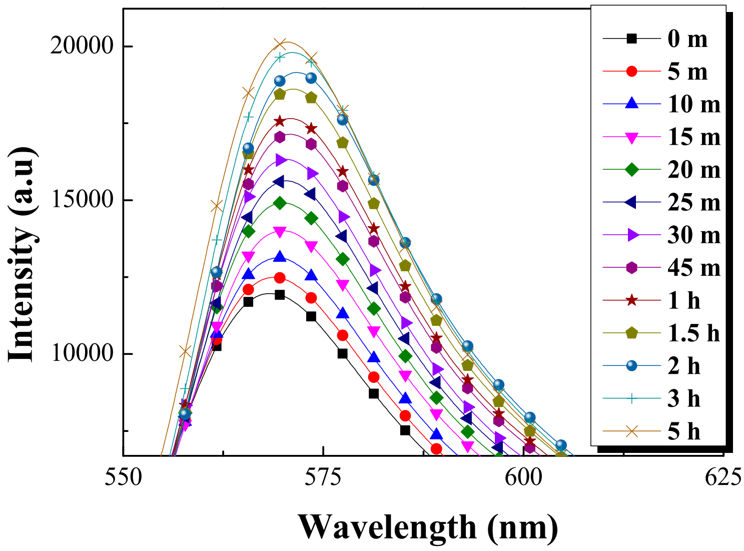

Figure S3. Variations of emission spectra of R-C/PLG-NCA ([R-C] = 5 x 10^{-3} M, [PLG-NCA] = 5 x 10^{-1} M) mixture solution in DMF over time. (λ_{ex} = 490 nm)

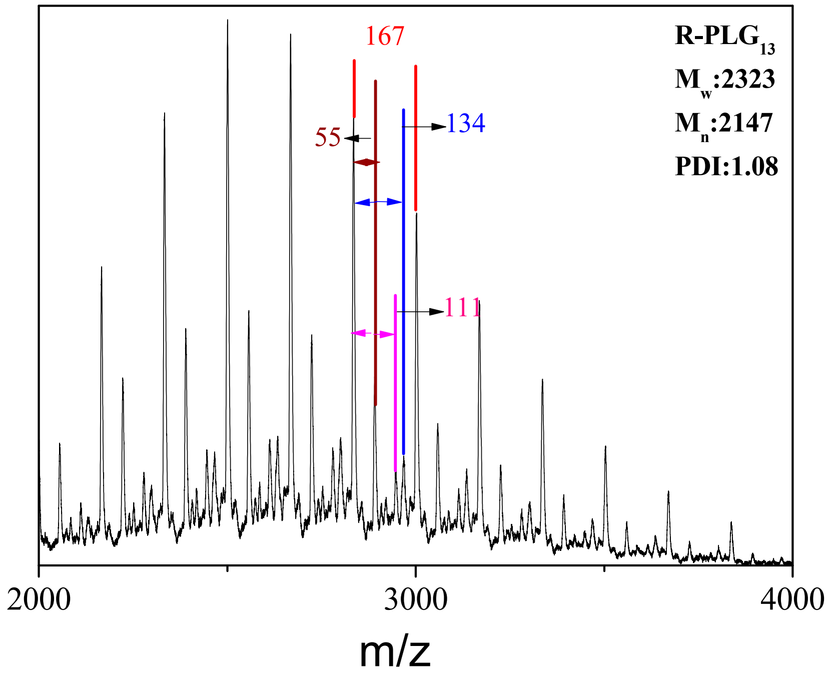

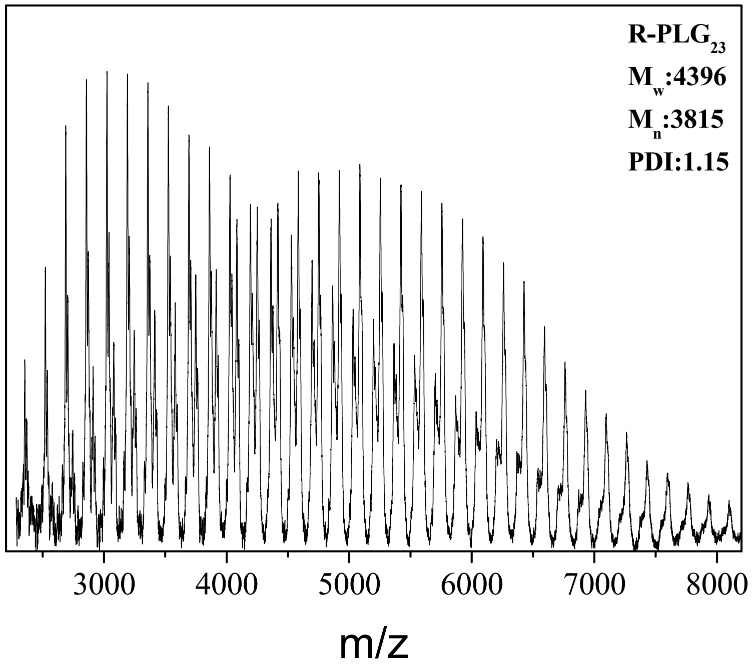

Figure S4. Madi-Tof mass spectra of R-PLG13 and R-PLG23.

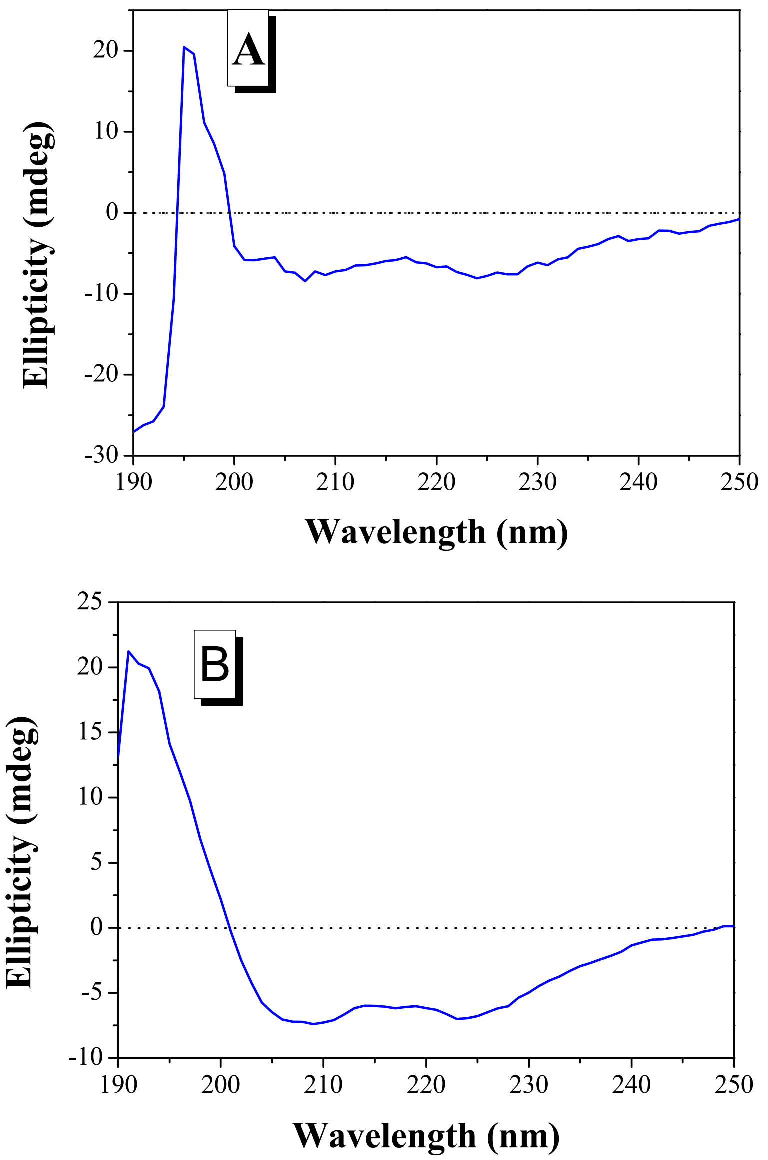

Figure S5. CD spectra of (a) R-PLG13 and (b) R-PLG23 in methanol.

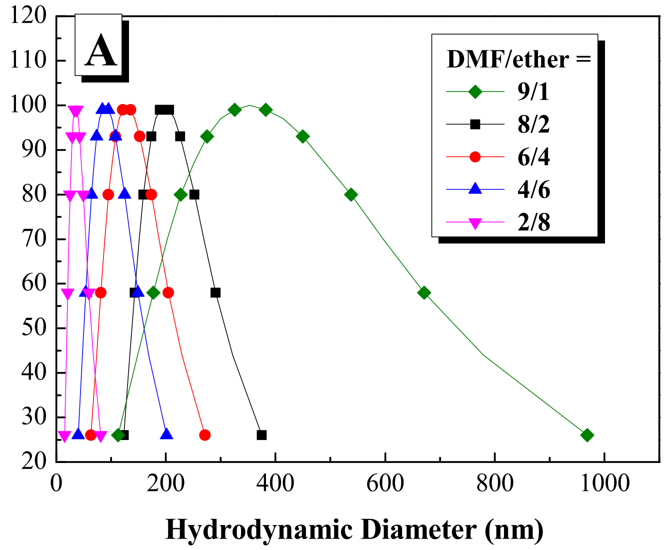

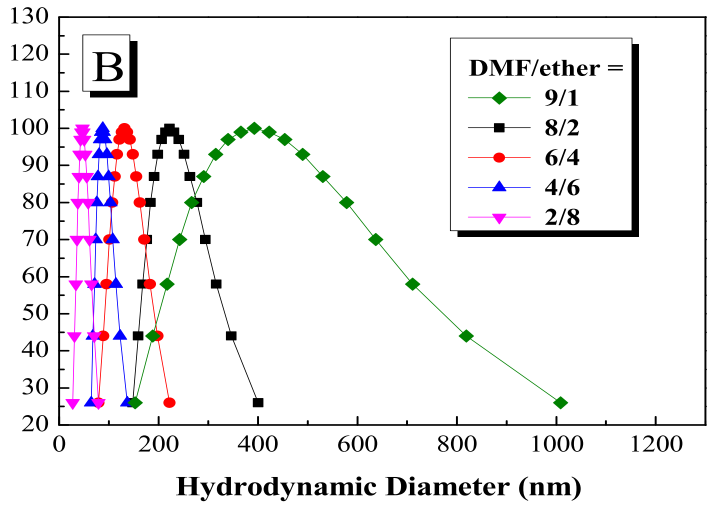

Figure S6. Particle sizes of (a) R-PLG$_{23}$ (10^{-4} M) and (b) R-PLG$_{13}$ (10^{-4} M) in solution mixtures of DMF/ether of different compositions.

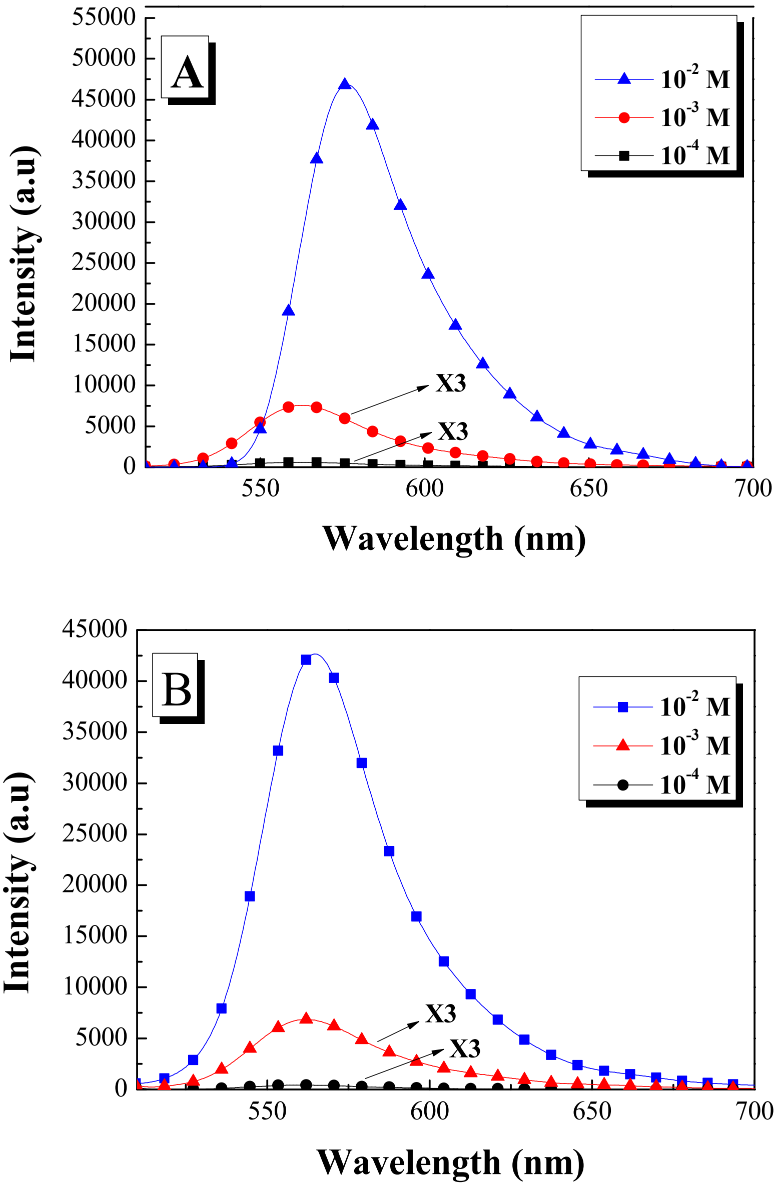

Figure S7. Emission spectra of (A) R-PLG$_{23}$ (10^{-4} M) and (B) R-PLG$_{13}$ (10^{-4} M) solutions in DMF of different concentrations (λ_{ex} = 490 nm).

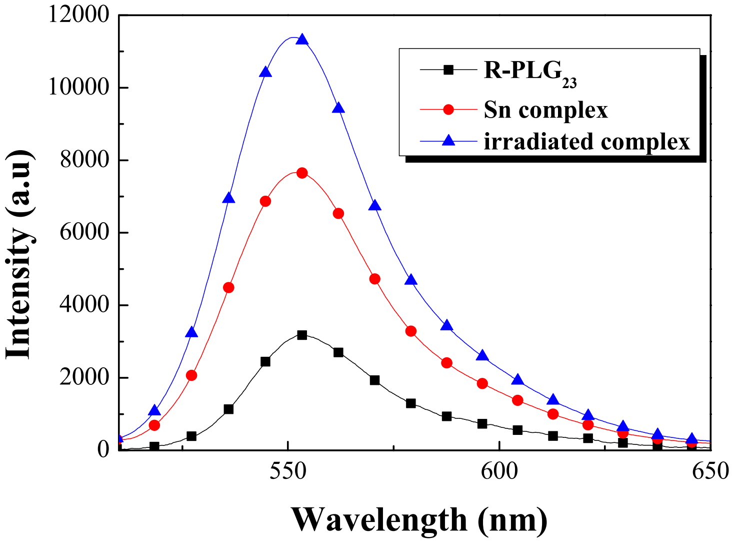

Figure S8. Solution emission spectra of R-PLG23, Sn(II) metal complex and the complex solution upon UV irradiation ([R-PLG23] = 10^{-4} M, [$SnCl_2$] = 10^{-3} M in THF, λ_{ex} = 490 nm)

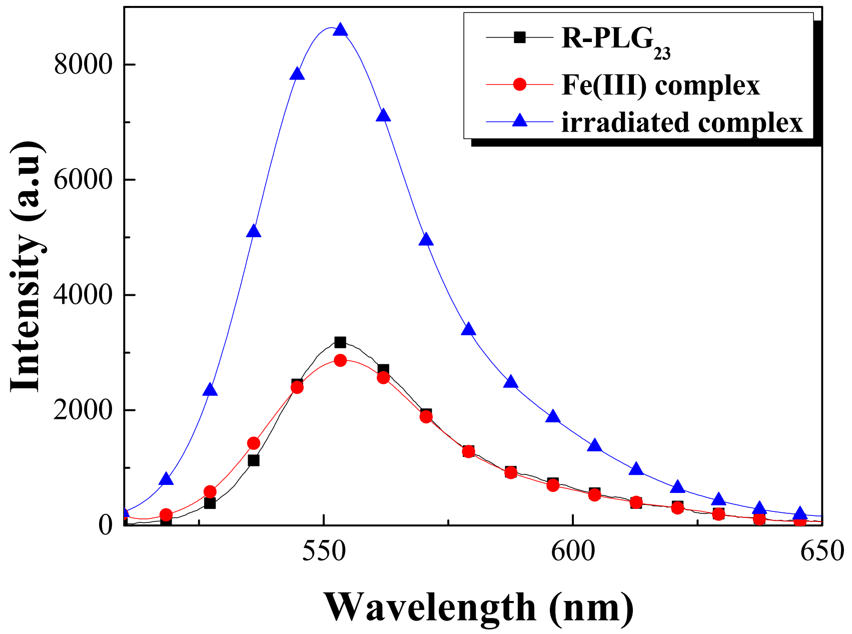

Figure S9. Solution emission spectra of R-PLG23, Fe(III) metal complex and the complex solution upon UV irradiation ([R-PLG23] = 10^{-4} M, $[FeCl_3]$ = 10^{-3} M in THF, λ_{ex} = 490 nm)
